# Scaling up medications for opioid use disorder in Kentucky: Qualitative perspectives of treatment organizations

**DOI:** 10.21203/rs.3.rs-5440415/v1

**Published:** 2024-12-12

**Authors:** Hannah K. Knudsen, Shaquita Andrews-Higgins, Sandra Back-Haddix, Michelle R. Lofwall, Laura Fanucchi, Sharon L. Walsh

**Affiliations:** University of Kentucky; University of Kentucky; University of Kentucky; University of Kentucky; University of Kentucky; University of Kentucky

**Keywords:** medication for opioid use disorder, treatment retention, staffing, community resources, policy, stigma

## Abstract

**Background:**

Underutilization of medications for opioid use disorder (MOUD), which reflects the limited number of patients initiating treatment and being retained in treatment, remains a persistent barrier to addressing the opioid epidemic. Using an adapted PRISM (Practical, Robust Implementation and Sustainability Model) framework, this study describes multi-level barriers and facilitators to expanding MOUD as part of the HEALing (Helping to End Addiction Long-term^®^) Communities Study in Kentucky (HCS-KY).

**Methods:**

Cross-sectional small group and individual semi-structured interviews were conducted with 60 employees representing 30 MOUD agencies in eight Kentucky counties from December 2022 to June 2023. A deductive-dominant approach to interviewing, with all interviews recorded and transcribed. Using a codebook informed by the adapted PRISM framework, a directed consensus-based approach to coding and thematic analysis was used.

**Results:**

Although some agencies had a fairly static number of patients, most described recent experiences with modestgrowth in MOUD census and the ability to provide same day/next day MOUD. Multi-level factors, includingorganizational, patient-level, and community characteristics and perspectives, were perceived to impact MOUDcensus. Organizational characteristics impacting growth included the physical space of the clinic and staffing. Organizational policies in some agencies constrained treatment retention, while other agencies implementedinnovations to better meet patients’ needs. Patients often encountered numerous challenges to treatmentinitiation and retention, including limited access to transportation, technology, safe and stable housing, and childcare. These patient-level barriers often reflected community characteristics, while community stigma alsoimpeded MOUD growth.

**Conclusions:**

These qualitative data revealed that some degree of growth in MOUD has occurred, but multi-level barriers are impeding further increases in treatment initiation and retention. Some barriers likely require policy changes related to financing and regulation, while other barriers require community-level efforts to decrease stigma and greater community investment in infrastructure, such as transportation and housing.

**Trial registration:**

ClinicalTrials.gov, NCT04111939. Registered 30 September 2019,

## Background

The opioid epidemic continues to be a major public health emergency in the United States (US), with fatal opioid-related overdose rates among the highest in the world [1]. In 2022, 81,806 individuals died from opioid overdose[2]. In recent years, there has been a marked increase in the fatal overdose rate among non-Hispanic Blackindividuals [3]. Opioid-related overdose mortality continues to have a profound impact, with an estimated 42.4%of US adults knowing at least one person who died by overdose and approximately 125 million US adults havingexperienced a loss due to overdose [4]. The economic costs of the opioid epidemic are vast, with the majority dueto reduced quality of life from opioid use disorder (OUD) ($390 billion) and the value of life lost due to fatal opioidoverdose ($480.7 billion) which accounts for over 85% of the total economic burden [5].

Medications that are effective for OUD (MOUD) and approved by the US Food and Drug Administration (FDA) include methadone (a full opioid-agonist), buprenorphine (a partial agonist), and naltrexone (an opioid antagonist) [6]. Treatment with buprenorphine or methadone is significantly associated with reduced risk of both overdose and recurrence of opioid use compared with no treatment or non-MOUD treatment [7, 8]. In addition to reducing mortality, buprenorphine and methadone increase quality of life and improve health outcomes [6].

Underutilization of MOUD remains a persistent barrier to addressing the opioid epidemic. Fewer than 30% of people with OUD in the past year actually receive MOUD [9, 10]. Buprenorphine and extended-release naltrexone can be prescribed in office-based medical practice, but until the Mainstreaming Addiction Treatment Act within the Consolidated Appropriations Act of 2023, buprenorphine prescribers were required to apply for a waiver from the Drug Enforcement Agency (DEA) with limits the number of patients they could treat, policies that were often considered barriers to adoption [11]. Historically, prescribers have had relatively small patient panels; among recent prescribers of buprenorphine, most have fewer than five buprenorphine patients [12]. Although buprenorphine utilization increased over the mid-2010s [13, 14], growth in the rate of buprenorphine initiation has largely been flat since 2018 [15]. Extended-release naltrexone reaches even fewer patients, approximately one-tenth of the number of buprenorphine patients [16].

In part, underutilization likely reflects the historical siloing of MOUD outside of traditional healthcare and the slow uptake of MOUD in the specialty SUD treatment sector. Of nearly 14,900 specialty SUD programs in the US, only about 47% provide buprenorphine, 42% provide extended-release naltrexone, and 14% are opioid treatment programs (OTPs) that are federally licensed to dispense methadone; as of March 2022, about 402,000 patients were receiving methadone, 258,000 were receiving buprenorphine, and 39,000 were receiving extended-release naltrexone [17].

Although MOUD underutilization has been well-documented in quantitative terms, relatively few recent qualitative studies have focused on the barriers to scaling up MOUD from the perspectives of specialty treatment programs that provide MOUD. To expand MOUD, it is critical to increase both the number of patients initiating treatment and the number retained treatment. Qualitative data from specialty MOUD providers may provide valuable insights into how to address the intertwined issues of MOUD initiation and retention.

Drawing on qualitative interviews with MOUD providers, this study describes multi-level barriers and facilitators to expanding MOUD in Kentucky. We draw upon an adapted PRISM (Practical, Robust Implementation and Sustainability Model) framework [18] from the HEALing (Helping to End Addiction Long-term^®^) Communities Study [19, 20] to elucidate the interplay between external context and internal context in understanding MOUD utilization. The external context represents the community in which an organization is located, including resources, policies, and community norms that may support or inhibit MOUD initiation and retention. The internal context represents both the MOUD organization itself as well as the people served by the organization. PRISM notes the importance of both characteristics (e.g., financial resources, staffing resources) and perspectives (e.g., organizational norms about growth, patient attitudes toward MOUD) within the internal context. Our goal is to apply the PRISM model to better understand the multi-level factors affecting MOUD initiation and retention, which has implications for scaling up MOUD.

## Methods

### Study design

This study was to prioritize evidence-based practices from the Opioid-overdose Reduction Continuum of Care Approach (ORCCA) for implementation [21–24]. Strategies to expand MOUD and improve retention represented ORCCA strategies. The HCS protocol (Pro00038088) was approved by Advarra Inc., the HCS’s single Institutional Review Board and was registered at ClinicalTrials.gov (NCT04111939).

### Data collection

Cross-sectional small group and individual semi-structured interviews were conducted with employees of MOUD agencies in eight Wave 2 HCS-KY counties from December 2022 to June 2023. Five counties were urban and three were rural. All counties all highly impacted by opioid overdose deaths, and each had a syringe service program, a jail, and at least one MOUD provider. If a county lacked a licensed OTP, a 50-mile radius was used to identify OTPs that may dispense methadone to the county’s residents. Through previous landscape analyses, relationships with agencies located in Wave 1 counties, and knowledge of local HCS staff, 39 MOUD agency locations were identified.

Recruitment involved email and telephone invitations. Of the 39 MOUD agencies, 77.0% (n = 30) participated in an interview, 12.8% refused (n = 5), and 10.3% (n = 4) were unresponsive to multiple attempts to contact them. A total of 27 interviews were conducted (as two interviews covered more than one location) with 60 individuals providing verbal informed consent and participating. Interviewers were trained HCS-KY Implementation Facilitators. All interviews were conducted prior to efforts to implement MOUD-related ORCCA strategies during the CTH intervention. Interviews were recorded and professionally transcribed. No payments were provided for participation.

The semi-structured interview guide included open-ended questions about MOUD census, resources needed to increase the patient census, barriers to MOUD access and retention, and the impact of the COVID-19 pandemic. Interview data regarding types of MOUD and current census differentiated types of organization and size. After the interview, participants were asked to provide demographic information via a five-question web survey.

### Data analysis

Similar to other qualitative studies in the HCS, a deductive-dominant approach to interviewing [25] and a directed thematic analysis approach to coding were used [26–28]. An initial codebook was created based on the PRISM framework, with code definitions and inclusion and exclusion criteria for each code. Similar to the process of Drainoni et al. [29], the coding team (HKK, SBH, SAH) used a consensus-based approach to apply the codebook to four transcripts; coding discrepancies and codebook updates were logged. Once consensus was reached, the remaining transcripts were independently coded in NVivo 12.0, with ongoing meetings to discuss any coding challenges. The codebook is included as Additional File 1.

After coding was completed, we conducted a thematic analysis [27, 28] of the multi-level factors impacting MOUD growth, including MOUD initiation and retention. To enhance rigor, the coding team reviewed code reports independently to identify themes, and then discussed the themes and representative passages during meetings until consensus was reached [30].

## Results

### Organizational characteristics

Of the 30 MOUD agencies, seven operated as OTPs, two delivered MOUD in outpatient primary care practices, and the remaining 21 were non-OTP specialty treatment organizations. Nine were located in rural counties. Regarding census, five agencies were very large (> 400 MOUD patients), nine were large (200–400 MOUDpatients), four were medium (100–199 MOUD patients), three were small (50–99 MOUD patients), and nine werevery small (< 50 MOUD patients).

#### Most organizations had experienced recent grown and had capacity for modest growth, but significant growth would stretch resources

Although some agencies had a fairly static number of patients, most described recent experiences with modest growth in patient census. For about half of the agencies experiencing growth, patient volume had increased at their existing sites with new admissions exceeding monthly discharges. For the other half of agencies experiencing growth, growth had occurred by the opening of new clinic sites or the purchase of existing clinics in the communities.

When asked about their perspectives regarding growth, nearly all participants indicated strong interest. In the words of one participant:

We are very interested. Anytime that we can get people into our program and of course help them, that’s our goal. We are increasing our providers. We recently brought on case management. And we’re looking to hire more counselors. So, it’s like the people are there and in place to serve. It’s just getting more patients here and building that client base, so.

(4346; very large, non-OTP) to growurban, was the ability

One indicator of capacity to grow was the ability to admit new patients quickly. Most agencies reported beingable to accommodate new admissions either with same day or next day admission appointments. As described byone participant:

We try to take them as soon as they walk in the door. Unfortunately, in this line of work, if you don’t take themwhen they’re ready for treatment or you try to schedule appointments, they usually generally will not come back. So, our goal is to get them in here to try to save their lives and not make them another statistic. So, we workreally hard if somebody walks in through the front door and wants to do an intake that we will scramble around ifwe have to find a counselor that can do it, because it is time consuming. But we will do whatever we have to do toget them in the same day.

(4247_4273_4274; large, urban, OTP)

Another participant described their capacity for new intakes similarly and noted that same day appointments were not the norm in other healthcare specialties:

I was just going to say with turnaround time, a patient cannot have an appointment, come to [clinic name] and leave that day with a counselor, with treatment, and with a case manager. All on the same day, without an appointment. It’s crazy. But it just honestly to me shows the dedication of [Participant 1] and the practice. We all roll the same way and put patients first. But to me that’s–I couldn’t do that at my doctor. I’m sure most people don’t have that luxury of, “oh, I’m sick today, can you get me in same day?” And we’re just like, “Just come, if you come, we’ll take care of you, just come.” And that’s kind of our motto throughout everything.

(4349_4348; very large urban non-OTP)

Of note, nearly all agencies described that patients received medication on the intake day.

Two other organizational characteristics likely to impact growth were the physical space of the clinic and staffing. For some, ample space was available to accommodate more patients but would require hiring additional staff:

Well, right now we, we, I would say we have unlimited capacity to grow. I mean, our biggest limitation is staffing, probably, more than anything.

(4157_4175_4176_4177; very large urban OTP)

Other agencies described that creative reallocation of space and renovations might support growth in their current location:

We have a group room that we only utilize for orientations twice a week, so there’s a group room there. We also have a couple offices that don’t have people in them yet, so there’s that. So, as we grow, and our census grows, there people in might bethose offices, but there’s not anybody in those offices now.

(4249; urban large OTP)

For other agencies, clinic space was a barrier to growth in that the total square footage of the current location could not accommodate an increase in patients and staff:

We would need a bigger clinic at some point. Ours is very small. Not much room for the current staff that we have there now. If we were doing 25 new patients a month, we’d probably outgrow that in six months easily.

(3824_4180_4173; medium, rural, non-OTP)

Thus, for some organizations the physical space of the clinic was adequate, only necessitating hiring more staff if the number of patients were to increase, while others would need to change locations if significant growth occurred.

Significant growth would necessitate hiring additional staff. Most agencies had enough staff for their current patients and current flow of new intakes but described staff as wearing “many hats”. It was quite normal for staff across the MOUD agency to carry responsibilities across multiple roles:

We’re a small office, so all my staff wears a lot of hats. My administrative assistant at the front desk right now is doing that insurance legwork for me. Like I said, before they’re even clients, he’s working with them before and trying to make sure that we have a place to send them.

(4156; very small, urban, non-OTP)

Another agency noted that prescribers were intentionally capped in terms of patient census so that prescribers could also provide other medical services needed by their MOUD patients:

We usually try to keep a nurse practitioner around 100 patients. Because we treat hepatitis C, we treat infectious disease and so they’ll manage other things like blood pressure. We’ll bridge script until we get patients into family doctors if they need to. We try to keep the census around 100 patients per provider.

(4211_4215; very small, urban, non-OTP)

Furthermore, significant growth would not only require additional prescribers of MOUD but also other professionals. Increased staffing that would be needed due to regulations for OTPs or regulations for residential treatment programs:

So, to increase that number by 25, right now our regulations read 40 patients per counselor. So, we would need enough to be counselors able to provide the care.

(4249; urban, large, OTP)

An agency providing MOUD in a residential setting also noted that challenges of hiring and retaining staff:

The primary thing we would need right now would be staff. And that’s not necessarily medical staff or provider staff, but clinical staff and direct supports. So that’s a big issue we’re seeing right now in terms of keeping our units staffed, especially overnight. We really try very hard to maintain an appropriate staff to client ratio because, obviously, we want to provide good care to the people that we have here with us. So that would be the primary barrier from our standpoint would be staffing.

(4196; small, urban, non-OTP)

For some agencies, hiring additional staff was viewed as challenging from a financial perspective. In the words of one agency*,“Yeah, so nurses. Nurses is our big one now and we just can’t afford them. We just can’t afford them.” (4333_4334, very large, urban, non-OTP)* In part, the financial challenges likely reflected market forces as nurses were in high demand due to the COVID-19 pandemic and wages were rising across the entire labor market. Another agency described wanting to build a partnership with the local jail to increase linkage to MOUD when people were released; such a partnership would lead to MOUD growth but, because linkage services were not billable, grant funding was needed but had not yet been obtained:

So, when they left the jail, they’d not only have familiar faces to go and continue on their road to recovery, [MOUD clinic name] faces, that they would have the Vivitrol^®^ already in their system and it would reduce their cravings once they hit the street again. So, it’s a re-entry specialist is this very specific job for this, and they do for about $70,000 a year. Probably a little bit more than that as a salary. So, neither the jail or [MOUD clinic name] could afford that. So, we keep waiting on these grants, and we have been part of two grants, hopefully for funding related to having a re-entry specialist. But we haven’t been successful in obtaining that.

(3824_4180_4173; medium, rural, non-OTP)

Financial constraints for some agencies thus limited their ability to begin new initiatives that would increase their census over time and better address unmet treatment needs.

### Organizational Policy and Innovations Served as Barriers and Facilitators to Growth

Organizational policy was sometimes viewed as an obstacle to growth, when policies were perceived to impederetention. Several MOUD agencies noted specific requirements within their treatment protocols, particularlyaround the frequency of office visits, that hindered retention:

We run a high intensity Suboxone program. We have a lot of requirements. We run, I call it a tight ship. We don’t play a lot with our Suboxone. We do have a high expectation. People who get Suboxone with us come daily for eight weeks, five days a week. And that’s a big thing when you’ve been running or living in the middle of addiction, and we expect a five day a week attendance.

(4156; very small, urban, non-OTP)

Additionally, counseling requirements, embedded within organizational policy, were described by several participants, which could make ongoing treatment more difficult for some patients.

Other MOUD agencies recognized that patients experienced multi-level barriers to retention and implemented innovations to reduce burdens on patients. Some innovations were billable to insurance, such as peer support, medical care for hepatitis C, or access to a psychiatrist. Other innovations, such as transportation assistance, were not billable which placed additional financial constraints on the agency:

So those patients are already fragile and that is 85–90% of our patient population. So, we immediately try and identify, are these going to be at-risk patients? Is their behavior showing us risky behavior? And so then, when we do the crisis management with these patients and we bring them in two to three times a week, we are providing transportation for them. So, we give them, that’s completely free. We can’t bill for that. This is something we’ve been doing since we moved to a bigger space. We kept seeing that transportation seemed to be the number one issue. That patients didn’t get to their appointment. And for some patients who did, unfortunately, they were being trafficked just so that they could have transportation to get to their appointments. So, we were like, “Okay, we have to stop this.”

(4333_4334; very large, urban, non-OTP)

Although transportation services were not billable to insurance, a small number of agencies had successfully competed for grant funding to provide transportation to the agency as well as other community locations. In one instance, participants described foregoing their own salaries to fund transportation services for patients. These varied strategies focused on building recovery support and addressing patients’ complex needs to reduce barriers to ongoing treatment and obstacles to recovery.

### Social Determinants of Health at the Patient-Level Affected MOUD Census

To some extent, constraints on growth occurred at the individual-level, with patients encountering numerous challenges to treatment initiation and retention, including transportation, communication/technology, housing, and childcare needs. Transportation barriers at the patient-level were common and frequently mentioned by agency staff. One agency discussed transportation in the context of patient discharges:

Probably over half of the discharges that we end up with are due to transportation issues. Whether it is not having transportation at all, or not being able to afford gas to get to and from.

(3820_3891_4209; very small, rural, non-OTP)

Having reliable transportation through a dependable vehicle or money to afford public transportation was a key factor in treatment initiation and retention. In some instances, patients lacking transportation resorted to walking long distances, even in unbearable conditions, to treatment in order to continue their journey of recovery as one agency acknowledged:

Participant 2: A lot of them walk.

Participant 1: We’ve had patients that walk from-

Participant 2: Four or five…

Participant 1: We have one that works, what? [Name] Road is how far out there?

Participant 2: It’s seven, eight…

Participant 1: 12 miles.

Participant 2: It’s 7, 8, 12 miles, they’re walking.

Participant 1: And they would leave their house at four o’clock in the morning to get here by what? 10:30? And have to walk all the way back. And they did that for a while until they just couldn’t do it anymore. And when winter was here, it was really brutal. So, a lot of our patients walk because they can’t afford.

(4247_4273_4274; large, urban, OTP)

Other participants shared that limited access to communication technology was also a significant barrier. Some patients lacked consistent access to cellular devices, preventin them from staying in regular contact with the treatment agency:

The problem is, number one, cell phones being able to get in touch with these patients. So, all of them have prepaid phones and they have these prepaid minutes. So, we send out reminders for appointments. So, if your appointment’s in a week and we can’t get a reminder to you, and you’ve used drugs within the time you were here last time to when you come in, it’s likely not that they’re going to remember they have an appointment. We have literally went to one of the patient’s houses because we hadn’t seen them in a while, and we were very worried about them and said, “Hey, please just come with us. We’ll get you some food, we’ll feed you at the office. Let’s just see what’s going on.” Right? Because we worry. So, our follow through is great, but the missing link is patients not having the access to be able to text or they’re out of their minutes and when they get here, the first thing they want to do was jump on our wi-fi because when they jump on our wi-fi, they can text mom, dad, or whoever. So yeah, because they have no minutes on their phones. Next to transportation, that is the biggest barrier that no one’s talking about.

(4333_4334; very large, urban, non-OTP)

Along with transportation and communication barriers, MOUD agencies served some patients who lacked safe, stable housing that was supportive of recovery. The housing environment an individual resides played a major role in their recovery journey, which was particularly challenging if the person lived with people who were actively using drugs. Such living situations increased the risk of relapse and affected treatment retention:

Because when they go back into that environment because of –housing is definitely one of the biggest issues. Because if they don’t have a safe housing, and they go back into the same environment, a lot of people relapse and then they stop coming

(4178; very small, urban, non-OTP)

Another participant discussed the importance of building a healthy support system in efforts to help patients feel connected to individuals who have shared understanding of addiction and commitment to recovery:

### Interviewer: Aside from transportation, what are some of the other significant barriers to treatment retention?

Participant: I think environment that they live in is probably the next biggest thing. They’re living with people who are all continuing to use, so eventually, that seeps into their heads and they just give up on treatment, because it’s easier to just use with the people that you’re around. They have nowhere else to go. They have to continue to live in these situations, or they don’t have anything outside of that that gives them any kind of push.

(3864_3926_4208; very large, rural, non-OTP) (dup: abstract ?)

In addition, women sometimes faced an additional barrier due to lack of childcare. One participant discussed the agency’s patient census was mainly women so childcare was a barrier:

We have mainly female patients here at [clinic location], and I don’t know if you all do this, but some type of childcare, I don’t know. Some people have to bring their kids into their appointments with them sometimes, and then sometimes they can’t come in because they don’t have somebody to watch their kids.

(4155_4194_4188_4210; very small, urban, non-OTP)

In summary, many patients experienced poverty, limiting their access to transportation, communicationtechnology, safe housing, and childcare. For these patients, MOUD retention was an ongoing challenge, whichconsequently impeded growth in the agency’s census.

### Community-Level Barriers to MOUD Growth: Local Stigma and Limited Infrastructure Constrained Growth

Barriers to MOUD initiation and retention at the patient-level reflected the community context. Regarding community culture, participants described that stigma against MOUD was often still prevalent. Patients often dealt with negative viewpoints from the general community, such as community perceptions towards different treatment options and harm reduction approaches. One participant shared what stigma towards MOUD treatment often looked like at the community-level:

But really breaking that negative stigma of, it’s horrible to say, but pill mill mentality. You hear treatment, people in small communities don’t hear, “Oh, we’re healing communities.” They hear, “Oh, you’re pushing scripts. Just take it out of their mouths. They just don’t need to do it again. Just stop using.” They think it’s really easy. Breaking stigma is a big thing.

(4211_4215; very small, urban, non-OTP)

A general sense of community stigma served to potentially undermine patients’ willingness to initiate and remain on MOUD.

Stigma could also be found in specific sectors of the community where MOUD patients experienced negative interactions as well as discriminatory organizational policies. One participant shared how stigma towards MOUD patients revealed itself in pharmacies, which prompted the agency to open an in-house pharmacy:

Going to the pharmacies locally was a big stigma because they weren’t treated very kindly picking up Suboxone scripts. Small towns talk. So [we] eliminated that barrier as well. They can come here, use the in-house pharmacy to pick up medications and avoid the pharmacy stigma hurdles.

(4211_4215; very small, urban, non-OTP)

Another participant shared how patients experienced stigma when navigating recovery support services such as sober living facilities:

So, a big issue that we see is actually after they’ve completed residential treatment… But one of the largest barriers we see is afterwards trying to refer clients to other sober living. It’s very often that transitional house or recovery house or halfway house, or however you want to phrase it, they don’t take people that are on Suboxone actively. So that’s a tricky issue that we have on the back end, and that’s probably the largest barrier that we have right now. You don’t want to set people up on a medication and then tell them, “By the way, the place that you’re going to, you can’t take this medication.” So, there’s often a shortage of quality options for them.

(4196; small, urban, non-OTP)

Communities often varied in terms of what resources they had, due to factors such as geographic location and size which impacted the community’s infrastructure. Transportation was often a barrier at the individual-level, but smaller rural communities generally lacked public transportation infrastructure and private transportation options (e.g., taxis, rideshare), which created a significant barrier at the community-level. In these communities, patients experienced substantial barriers to intake and ongoing MOUD appointments as well as recovery support services:

Community resources are huge. We just don’t have a whole lot to refer people out to. If they don’t have transportation and they can’t ride the [local van service], then there’s nothing here that we can refer them to, so being in a small community kind of hinders us in that way.

(3864_3926_4208; very large, rural, non-OTP)

Another participant shared how there was no transportation access at all in their community which led to patients without a vehicle either walking to their appointment or depending on someone in their support system to drop them off:

There is no bus service, there’s no cabs, there’s nothing here. They’ll walk or get dropped off by someone they know.

(3824_4180_4173; medium, urban, non-OTP)

Even in communities with public transportation, schedule mismatches and limited service routes remainedsignificant barriers, particularly among those who are employed:

…the bus system is difficult for people that have to work, you know, it takes multiple hours to get to us and thenback home and whatever, so it’s not really a feasible option for a lot of patients. So, transportation I would say is probably number one.

(4157_4175_4176_4177; very large, urban, OTP)

At the community-level, transportation systems often failed to meet the needs of MOUD patients, with smaller, rural communities lacking any public transportation infrastructure and more urban communities having public transportation systems that were not designed to efficiently move people to their destinations.

Smaller, more rural communities experienced technological infrastructure barriers, which made it more difficult to expand MOUD treatment in the era of telehealth. These communities continued to have limited access to broadband internet and cell reception, which impeded agencies attempting to mitigate patients’ transportation barriers by implementing telehealth. One participant noted*,“And Kentucky’s the worst part because we have deserts for internet, period.” (4333_4334; very large, urban, non-OTP)*

Housing barriers also reflected community-level resources. One specific resource was transitional and recovery housing services. Although recovery housing was limited in most communities, there were more transitional housing options for men and fewer options for women, particularly for pregnant women or women with children. One participant shared:

Another issue that we do have in-terms of --- sorry to circle back to barriers, just housing in general is a major barrier depending on --- it’s a lot easier for men to find transitional housing than it is for women to find transitional housing, especially women and children. So that’s a major focus right now for us is trying to acquire more housing resources for our female clients, especially our female clients that have their children and are currently providing care for their children.

(4196; small, urban, non-OTP)

In summary, the local context constrained MOUD growth though stigma and limited infrastructure, exacerbatingpatient-level barriers.

### Broader External Factors Impacting Growth: Insurance-RelatedPolicies and the COVID-19 Pandemic

Insurance-related policies impacted MOUD growth in multiple ways. First, some new patients needed to acquire insurance after incarceration or other life circumstances had resulted in a coverage lapse:

A lot of times insurance is a barrier. We have a lot of people who call, they want treatment, but they’ve been incarcerated and their Medicaid lapsed, or they’ve been in these streets and just forgot to send in some paperwork and their Medicaid lapsed. So, when those patients come in, the first barrier we have to get through, is getting them insurance so they can get their medication and get their doctor’s visit. So, a lot of times getting your insurance reinstated is a hurdle.

(4156; very small, urban, non-OTP)

This type of insurance barrier was surmountable, as Kentucky is a Medicaid expansion state, so agency staffwould help patients obtain this type of insurance. Other insurance-related barriers, such as deductibles and co-pays in commercial insurance plans, were more difficult to overcome:

So, some of them could be the commercial health insurance, a lot of the plans require them to pay their fulldeductible before they cover. So, Anthem is an example. Their copay is $20 a day. Well, our cash fee is only $15 aday. So, in order for them to utilize their Anthem insurance, they have to pay their full $2000, $3000, $5,000deductible, whatever it may be, before they could actually utilize that benefit. So, for a lot of our patients, theychoose not to utilize the insurance benefit unless they’ve got some other health concerns, too. Because they’repaying $5 a day more than if they were just a self-pay patient

(4181_4054_4159, small, rural OTP)

Finally, many participants noted that the policies of Medicaid’s transportation service resulted in barriers to MOUD retention. Medicab’s 72-hour advance scheduling policy was difficult for patients to navigate. Other Medicaid policies rendered some patients as ineligible to use the service. The following participant described multiple policy-related obstacles to using Medicaid transportation:

We do have women who use the Medicaid transportation. But again, that takes planning and early in recovery, that’s hard to plan 72 hours in advance. And oftentimes they’re not reliable, the Medicaid transportation. So, if that’s our last option, that we use. But that also can run into barriers because you can’t live within a mile of a [bus] stop. You can’t have a car registered to your name. So even that resource can become challenging.

(4189; very small, urban, non-OTP)

A second broader external factor affecting MOUD agencies were policy changes enacted during the COVID-19 pandemic that impacted treatment access and retention, and hence, the agency’s MOUD census. Some described positive impacts on retention due to the rapid rise in telehealth, but also negative impacts in terms of the stresses of the pandemic on patients:

I guess first thing I would say around positives, I’ll start there, is just that we’ve made better utilization of telehealth services and electronic means of outreach to patients. It’s helped with patient engagement and retention as well, as people are able to be seen via telehealth and not just in office all of the time. Then some of the negatives that I would say, we’ve seen an increased amount of mental health needs with our patient population, increased anxiety and depression for sure, obviously increased risk of overdose and in some cases overdose.

(4167_4164_4191_4161_4192_4190; large, urban, non-OTP)

Others noted that although telehealth had maintained access to MOUD during the height of the pandemic, there were some challenges, such as increased competition with telehealth-only MOUD agencies and a minority of patients not wanting to return to in-person care. These impacts may not have been large in terms of retention but were notable:

Participant: We never offered telehealth services prior to the COVID-19 pandemic, and now that is more of an option for our patients, so I think that’s a positive way that it’s impacted us. I do think the year that most things were telehealth kind of disengaged a lot of patients.

Interviewer: When you say disengaged--

Participant: They started in that time, they got used to doing telehealth and not coming into the clinic, so when we tried to transition back to more in-house services, we lost a lot of patients, because they got used to the telehealth and it was more convenient for them…I think that the pandemic opened up a lot of options for fully telehealth clinics, and that has impacted our retention to, not a huge extent, but we’ve lost maybe five or ten patients to strictly telehealth clinics, because it’s easier for them. I would say not a huge impact on us, but five or ten patients is five or ten patients. You want to keep as many as you can engaged in treatment.

(3864_3926_4208; very large, rural, non-OTP)

For OTPs, the pandemic resulted in changes to federal regulations that allowed for telehealth and greater flexibility in take-home medications. Two OTP participants described how COVID-19 impacted their organization and the broader regulatory environment:

Participant 1: I mean, candidly, I think it [the pandemic] opened up a lot of extra doors and because primarily before we didn’t use telehealth for anything. We didn’t use it for sessions, we didn’t use it for anything. So, I think at least for a temporary piece, it opened our door, our eyes opened to, we can think outside the box, and we did that. We were able to expand take-homes to our patients and work within different parameters. So, I think we really were forced to look outside the box. And I think…we as an agency and as a field kind of grew from that. We made mistakes along the way obviously, but I think we learned a lot about individualized treatment even more so than we were already doing.

Participant 2: Yeah. I think it also forced federal and state regulators to, I think it really forced them to think outside the box quickly and it proved to them that we could handle treating a whole lot of patients in a crisis crazy situation and could really take care of our patients really well, and the patients could also handle it. And it’s resulted in, not only have they continued to extend a lot of the COVID exceptions … currently our state regulations are being amended. And at the federal level, the DEA and SAMSHA are expected to make the COVID exceptions permanent in their regulations. But this is something that has several of those things are, knock on wood, it looks like they’re going to turn out working to our and the patient’s advantage.

(4181_4054_4159; small, rural, OTP)

The likely permanence of these regulatory challenges was viewed by most participants from OTPs as opportunities to maintain greater flexibility and the ability to better meet the needs of patients, thus supporting efforts to retain individuals in care.

## Discussion

Expanding the number of people receiving MOUD is an important strategy for addressing the opioid epidemic, but qualitative interviews with MOUD agency staff revealed the multi-level factors that likely affect MOUD census. Agencies largely had the organizational resources to support modest levels of growth, but large-scale growth would likely strain staffing resources and may exceed the clinic’s physical space. Additional constraints on growth included organizational policies, patient-level characteristics, limited community resources, and stigma.

Of note, many programs had experienced recent modest growth in their census, either through growth in existing locations or the establishment of new locations. Some growth may have resulted from individuals returning to care after the COVID-19 pandemic had waned, but this growth may also reflect the persistently high rates of OUD in Kentucky [31]. Structural changes in healthcare financing may have also supported growth. Kentucky expanded Medicaid under the Affordable Care Act, and among people who use drugs, the increase in health insurance has been dramatic, with a study of a longitudinal cohort of people who use drugs finding an increase from 34% in 2008 to 87% in 2017 [32]. In July 2019, Kentucky’s Medicaid program finally provided coverage for methadone dispensed by OTPs; by mid-2021, about 60% of individuals receiving methadone in Kentucky were Medicaid beneficiaries [33]. These structural changes are important given that cost of MOUD has been cited as a patient-level barrier to entering care [34]. However, even with these policy changes, some patients continue to encounter barriers related to insurance. For example, in April 2020, Kentucky Medicaid removed the prior authorization requirement for long-acting injectable buprenorphine, and while this was associated with increased utilization, some Medicaid managed care organizations continued to process denials for this medication [35].

Another notable finding regarding capacity for growth is that nearly all participants described models of intake that provided MOUD on the same day or day following intake. Waitlists to MOUD have long been noted in the literature [34, 36], so the rise of same day/next day MOUD represents a substantial improvement in treatment access. Same day/next day intakes are central to low-threshold treatment models that have been described as a means to expand MOUD [37, 38]. Many participants drew the connection between same day/next day MOUD and harm reduction, particularly in reducing the risk of overdose, which also aligns with the core components of low-threshold care [38]. Other aspects of low-threshold care were not described, particularly regarding counseling requirements which can be detrimental to retention [39]. In part, Kentucky’s regulatory environment may prevent the loosening of counseling requirements; an analysis state-level regulations for methadone categorized Kentucky as “high restrictiveness on patient experience,” which was characterized by set schedules for counseling [40].

State regulations pre-pandemic also set schedules for buprenorphine office visits and for counseling [41, 42]. As of March 2023, a policy analysis showed Kentucky was one of 19 states explicitly regulating buprenorphine [43]. Buprenorphine regulations promulgated by the Kentucky Board of Medical Licensure (KBML) specify need for behavioral modification, frequency of clinician visits and drug testing, initiation doses and maintenance dose maximums, and this June, the KBML proposed revising its regulations to become more stringent (e.g., removing allowances for split dosing, requiring gabapentin testing) [44, 45]. By many other measures, policy leaders in the state have taken proactive steps to make methadone and buprenorphine more accessible (e.g., Medicaid expansion, Medicaid covers all the FDA-approved MOUDs). This highlights the complex external context that affects MOUD treatment access and the simultaneous need for active participation from community, state, and national stakeholders to try to prevent implementation of proposed access-limiting regulations.

The ability of MOUD agencies to grow, particularly through increased retention, was constrained by multiple factors. A key organizational constraint on growth was staffing. Previous research suggests that achieving growth in MOUD prescribers requires intentional strategies, such as having someone dedicated to the role of recruitment and having a specific budget allocation for recruitment, yet these strategies are not widely used [46]. Recruitment of other professions, such as counselors, may be particularly challenging in rural areas [47]. Furthermore, the treatment field has long experienced elevated rates of staff turnover [48, 49]. These challenges were likely exacerbated by the COVID-19 pandemic; recent research has documented an increasing percentage of the healthcare workforce transitioning to non-healthcare sectors [50].

Constraints on growth were also described at the patient-level and community-level. Many MOUD patients face multi-level challenges related to the social determinants of health, such as lack of transportation and housing [36]. Participants underscored lack of transportation resources as a key barrier, which has been highlighted in qualitative interviews [51] and surveys with MOUD patients [52, 53]. However, it is important to recognize that transportation barriers are not solely a patient-level phenomenon, but rather reflect community infrastructure (i.e., the absence of public transportation entirely or time-intensive transportation systems). Participants noted that many patients faced housing challenges such as homelessness, unsafe housing situations, and recovery housing that was hostile to MOUD. Similar to our study findings, a qualitative study of patients and providers identified lack of safe and stable housing as a major barrier, interfering with attending appointments and increasing risk of relapse [54]. The issue of housing again highlights the intersection of the personal and the structural, with broader economic forces resulting in the lack of affordable housing posing a significant threat to individual and population-level health [55].

Many participants shared how community stigma impacted the patients that they serve. Stigma was often noted as one of the major barriers impacting MOUD growth, and it operated across multiple levels, both inside and outside, of the MOUD treatment community. These findings on stigma surrounding MOUD growth are consistent with recent qualitative studies [56–59]. Similar to our findings, other studies have shown how MOUD-related stigma is a significant factor in the types of MOUD treatment that patients chose and their experience in MOUD treatment [34, 57, 58]. A clear pattern emerged in our findings beyond general community stigma, with stigma operating in other health-related settings, such as pharmacies and recovery housing, that negatively impacted patients receiving MOUD.

Several limitations should be noted. First, these interviews were conducted with MOUD agencies in a single state, so the themes identified may not generalize to other states. Second, participants largely worked within specialty treatment settings where OUD or SUD treatment was the primary focus as opposed to settings where MOUD has been integrated into outpatient medical care. Third, the interviews were conducted in the context of establishing partnerships for the HCS, which may have impacted how participants responded. Finally, we were unable to conduct qualitative interviews with MOUD patients, given the broader design of the HCS, but recognize that patients’ perspectives may differ from staff working in MOUD agencies.

## Conclusions

There is an ongoing need to scale up MOUD to meet the needs of people with OUD through increasing access and improving retention. These qualitative data from interviews with MOUD agencies revealed that some degree of growth has occurred, but multi-level barriers are impeding further improvements in treatment initiation and retention. Some barriers could be addressed through policy changes related to how MOUD treatment is financed and regulated, while other barriers would require greater community investment in infrastructure, such as innovative strategies to improve transportation and housing. Future research is needed to better understand the impacts of addressing the social determinants of health in the context of MOUD initiation and retention.

## Data Availability

Data reported in the current study are not publicly available to protect the privacy of organizational partners.

## Abbreviations

CTH: Communities That HEAL
DEA: *Drug Enforcement Agency*
EBP: Evidence-based practice
FDA: *Food and Drug Administration*
HCS: HEALing (Helping to End Addiction Long-term^®^) Communities Study
HCS-KY: HEALing (Helping to End Addiction Long-term^®^) Communities Study Kentucky site
HEAL: *Helping to End Addiction Long-Term*
KBML: Kentucky Board of Medical Licensure
MOUD: *Medication for opioid use disorder*
ORCCA: Opioid-overdose Reduction Continuum of Care Approach
OTP: *Opioid treatment program*
OUD: Opioid use disorder
PRISM: Practical, Robust Implementation and Sustainability Model
SAMHSA: Substance Abuse and Mental Health Services Administration
US: United States
